# Huber Proprioceptive Training and Alpha-Lipoic Acid: A New Approach in Optimizing Nerve Function in Patients With Chronic Low Back Pain

**DOI:** 10.7759/cureus.105235

**Published:** 2026-03-14

**Authors:** Aldijana Kadrić, Emina Rovčanin, Damir Čelik, Dževad Vrabac, Đemil Omerović, Ena Gogić, Amir Merdović, Selma Šabanagić-Hajrić, Edina Tanović

**Affiliations:** 1 Physical Medicine and Rehabilitation, Clinical Center of the University of Sarajevo, Sarajevo, BIH; 2 Pharmacy, Public Institution "Apoteke Sarajevo", Sarajevo, BIH; 3 Physical Medicine and Rehabilitation, Clinical Center University of Sarajevo, Sarajevo, BIH; 4 Orthopedics and Traumatology, Clinical Center University of Sarajevo, Sarajevo, BIH; 5 Neurology, Clinical Center University of Sarajevo, Sarajevo, BIH

**Keywords:** alpha-lipoic acid, chronic low back pain, electromyoneurographic activity, proprioceptive system, sensorimotor training

## Abstract

Background

While there is evidence supporting the potential of alpha-lipoic acid (ALA) to improve nerve conduction parameters in certain neurological conditions, particularly diabetic neuropathy, its specific effects on nerve conduction in chronic low back pain (CLBP) patients have not been directly addressed in the current context. There is a connection between proprioceptive system deficits and movement control dysfunction in patients with chronic lower back pain, but the exact mechanism of this link is unknown.

Aim

To analyze the effectiveness of the proprioceptive rehabilitation method using the Huber system of exercises and to evaluate nerve conduction study findings in a patient with CLBP treated with ALA.

Methods

This prospective quasi-experimental clinical pilot study with a pre-test/post-test repeated-measures design included 15 patients treated at the Clinic for Physical Medicine and Rehabilitation, Clinical Center of the University of Sarajevo, during a three-week follow-up period. The study was conducted between January 1, 2025, and April 30, 2025. The patients were treated with 600 mg of ALA supplementation per day and participated in Huber proprioception training five days per week. Patients were followed for the next three weeks, with two study visits: one at baseline and one at the end of the study. The study visits included electromyography (EMG) and nerve conduction studies. A p-value of <0.05 was considered significant.

Results

Out of the total patients (n=15), 11 (73.3%) were male. The most commonly affected levels were L4/L5 (13, 87%), followed by L3/L4 (1, 6.5%) and L5/S1 (1, 6.5%). There was a statistically significant median increase after three weeks in both proximal (z=-3.298, p<0.001) and distal peroneal M wave amplitude (z=-3.415, p<0.001). A statistically significant median increase was also observed in proximal (z=-3.408, p<0.001) and distal tibial M wave amplitude (z=-3.409, p<0.001).

Conclusion

Future research with a larger sample size and longer follow-up periods is needed to evaluate the long-term effects of the sensoriomotor training program combined with ALA supplementation in patients with chronic low back pain.

## Introduction

Chronic low back pain (CLBP) is a common and difficult health issue that is becoming more widespread around the world [1]. Studies show that 60 to 80% of adults will have low back pain at least once in their lives [1,2]. For many, the pain lasts more than 12 weeks and becomes a chronic problem [2]. This makes chronic low back pain a leading cause of disability, affecting quality of life and creating economic challenges for individuals [3]. The high cost of treatment puts a direct financial strain on patients, while the condition also lowers work capacity, leads to longer absences from work, and results in a loss of workers. Because of these effects, chronic low back pain is a major public health concern [4].

The causes and mechanisms of low back pain are complex and multifactorial. It is believed that a combination of biomechanical, neurological, psychological, and social factors are at the root of it [5]. Such a complex etiopathogenesis makes this condition complex to diagnose and establish effective treatment. An important role in the etiology is played by the proprioceptive system, which allows our body to perceive the sense of body position in space, which is crucial for maintaining stability and coordination of movements [6]. Disturbed proprioception results in impaired motor function and reduced spinal stability, which ultimately increases the risk of injury and further increases the pain in this condition [7].

The connection between impaired proprioception and chronic back pain has attracted increasing attention among researchers [8]. Previous studies have shown that patients with chronic back pain regularly have reduced ability to perceive and control movement, which can further prolong and worsen existing symptoms [9]. However, the mechanism of the connection between these two conditions has not been fully elucidated, and additional research on this topic is needed to better and more accurately understand the pathophysiological mechanism of the connection between these two conditions, as well as the neurophysiological changes, all with the aim of creating the best possible rehabilitation protocol for patients [5].

Alpha-lipoic acid (ALA) is a potent antioxidant and a coenzyme involved in cellular energy metabolism [10]. Its ability to reduce oxidative stress and inflammatory processes has made it the subject of numerous studies in the field of neurological disorders, particularly diabetic neuropathy, where it has demonstrated a positive effect on nerve function and the reduction of neuropathic pain [10]. Although the efficacy of ALA in neuroprotective and neuroregenerative processes has been established in certain conditions, its application in the context of chronic low back pain, as well as in combination with proprioceptive rehabilitation, has not been sufficiently investigated or clearly defined [11]. 

Rehabilitation programs that incorporate proprioceptive training, such as the Huber exercise system, focus on improving sensorimotor integration and neuromuscular coordination [12]. This system employs specific exercises on a device that enables controlled movements and proprioceptive stimulation, aiming to enhance functional stability and reduce pain symptoms [13]. There is evidence suggesting that early and systematic application of such therapeutic modalities may contribute to better management of CLBP symptoms; however, there is a lack of rigorous studies that quantitatively assess these effects, particularly in combination with ALA supplementation [14].

The combination of pharmacological intervention, such as ALA supplementation, and targeted proprioceptive rehabilitation may offer an innovative, integrative approach to the treatment of CLBP [15]. The potential synergistic effects of these methods may lead to improved neurophysiological outcomes, reduced pain intensity, enhanced functional capacity, and better quality of life for patients [14]. Considering the complex and multifactorial nature of CLBP, such a multidisciplinary approach is essential for the development of effective, individualized therapeutic protocols [11]. 

This study aims to analyze changes in nerve conduction and functional outcomes in patients with chronic low back pain through the application of proprioceptive rehabilitation using the Huber system in combination with ALA supplementation. The results may contribute to a broader understanding of the neurophysiological effects of integrative therapy and open new avenues for further research and clinical practice in the global context of chronic pain management.

Electromyoneurography (EMNG) is an electrophysiological method that measures action potentials in muscles using needle or surface electrodes. It can demonstrate damage to the axons of motor fibers. This method can also determine whether there are signs of neurogenic damage in the tested muscles, whether the neurogenic damage originates from a peripheral nerve, plexus, or spinal root, whether the damage is acute or chronic, and the degree of damage. Additionally, it can help identify which spinal roots are involved in the neurogenic damage. EMNG is the "golden standard" in diagnosing disorders within the peripheral nervous system. This study was previously presented as an abstract at the 16th Physical and Rehabilitation Medicine Mediterranean Congress, held September 18-21, 2025, in Šibenik, Croatia

## Materials and methods

This prospective quasi-experimental clinical pilot study with a pre-test/post-test repeated- measures design included 15 patients treated at the Clinic for Physical Medicine and Rehabilitation, Clinical Center of the University of Sarajevo, during a three-week follow-up period. The study was conducted between January 1, 2025, and April 30, 2025. Inclusion criteria were: MRI- confirmed lesion of the sensorimotor segment of the lumbar spine (protrusion or extrusion disc, foraminal or central stenosis, nerve root compression), age >18 years, and signed informed consent. Exclusion criteria included non-compliance with the study protocol and refusal to participate.

After providing written informed consent, all participants underwent physical examination, clinical assessment, and electromyoneurography (EMNG) analysis. Basic demographic data (age, sex, body weight, and height) were collected at baseline. Study visits included electromyography (EMG) and nerve conduction studies. EMNG analysis was performed at baseline and after 21 days of participation in the rehabilitation program. Participants received 600 mg of alpha-lipoic acid supplementation daily and underwent Huber proprioceptive training five days per week. Participants underwent Huber® 360 MD proprioceptive training (Proxomed, Schnaittach, Germany) five times per week for three weeks, with each session lasting 30 minutes. The program included balance, postural control, and core stabilization exercises, adjusted individually to each patient's abilities. Exercise intensity was progressively increased according to patient performance throughout the study period.

Patients were followed for three weeks, with two study visits: baseline and end-of-study evaluation.

Electromyoneurography (EMNG) assessments were performed at baseline and after 21 days using a Synergy EMG system (model CZC6190VV7, Hewlett-Packard, Palo Alto, California). Nerve conduction studies of the tibial and peroneal nerves and needle EMG of selected lower limb muscles were conducted according to standardized procedures, including measurements of latency, amplitude, and conduction velocity. Although latency and conduction velocity were also recorded, they were not selected as primary outcomes because they are less sensitive to short-term intervention effects. M-wave amplitude was chosen as the primary outcome due to its responsiveness to changes in motor unit recruitment and neuromuscular excitability.

As this was a pilot study, no formal power-based sample size calculation was performed. A convenience sample of 15 participants was enrolled to assess feasibility and to obtain preliminary estimates of variability and effect magnitude to inform the sample size calculation for a future definitive trial. Statistical analysis was performed using SPSS (version 28; IBM Inc., Armonk, New York). Continuous variables were assessed for normality using the Shapiro-Wilk test. As electrophysiological parameters demonstrated non-normal distribution and the sample size was small (n=15), non-parametric methods were applied. Changes between baseline and three-week follow-up were evaluated using the Wilcoxon signed-rank test. Effect sizes were calculated as r = Z / √N, where Z represents the Wilcoxon test statistic and N the total number of observations.

## Results

A total of n=15 patients were included in the study. The majority were male (n=11, 73.3%), while four participants (26.7%) were female. The median age of female participants was 44 years (IQR 43-49.5), compared to 34 years (IQR 34-48) in males. No statistically significant sex-related difference in age distribution was observed (p=0.133), indicating comparable baseline characteristics between groups (Table 1).

**Table 1:** Sociodemographic characteristics of the participants and between-sex comparisons Values are presented as n (%) or median (IQR); ¹ Binomial test vs. 50%; ² Mann–Whitney U test (U=10.500, z=-1.504)

Variable	Females	Males	p-value
n (%)	4 (26.7)	11 (73.3)	0.118 ^1^
Age (years)	44 (43-49.5)	34 (34-48)	0.133 ^2^

The most frequently affected level was L4/L5 (13, 87%), followed by L3/L4 (1, 6.5%) and L5/S1 (1, 6.5%), demonstrating a predominance of L4/L5 involvement within the cohort.

**Figure 1:**
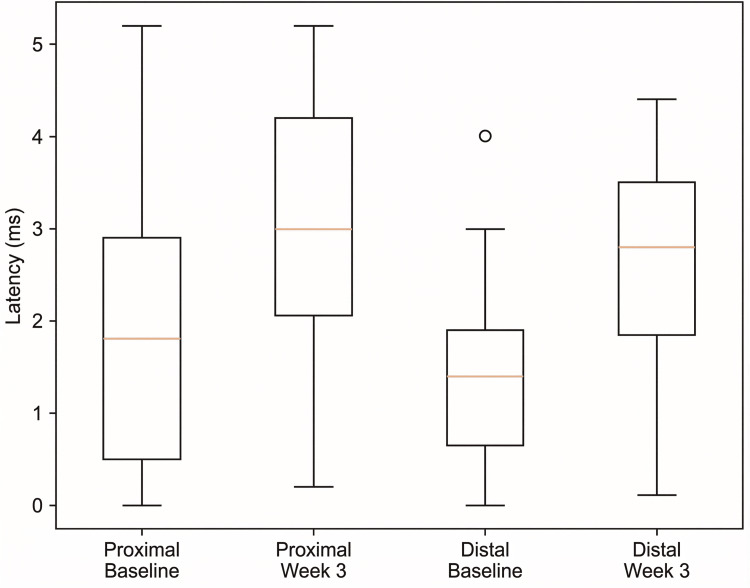
Changes in peroneal nerve M-wave amplitude after 3 weeks

After three weeks, a statistically significant increase in M-wave amplitude was observed in the peroneal nerve at both proximal (z=-3.298, p<0.001) and distal stimulation sites (z=-3.415, p<0.001) (Figure 1).

The magnitude of change was large for both stimulation points (r = 0.85-0.88), indicating a substantial electrophysiological improvement. The slightly higher effect size observed at the distal site suggests a consistent and pronounced recovery pattern across recording locations.

These findings demonstrate a robust response to the intervention, reflected not only by statistical significance but also by clinically meaningful effect sizes.

**Figure 2:**
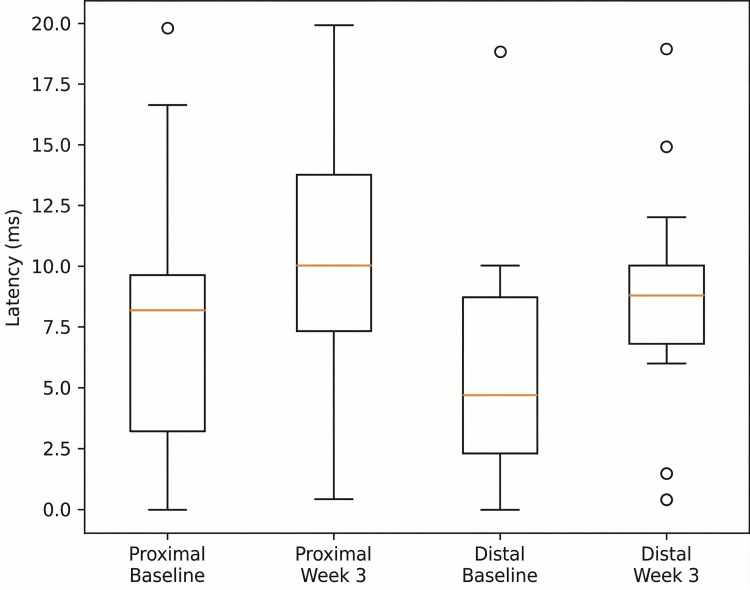
Changes in tibial nerve M-wave amplitude after three weeks

Similarly, a statistically significant increase in tibial nerve M-wave amplitude was detected at proximal (z=-3.408, p<0.001) and distal sites (z=-3.409, p<0.001) after three weeks (Figure 2).

Effect sizes were consistently large (r=0.88), confirming a strong magnitude of neurophysiological improvement. The uniformity of effect size across proximal and distal measurements suggests a stable and generalized enhancement of tibial nerve function.

When comparing the two nerves, both the peroneal and tibial nerves demonstrated similarly large effect sizes, indicating comparable responsiveness to the intervention. Distal stimulation sites tended to show marginally higher effect magnitudes, although the overall pattern of improvement was consistent across all parameters.

Despite the relatively small sample size, the large effect sizes support the clinical relevance of the observed electrophysiological changes.

The calculated effect sizes (r) indicated a large magnitude of change for both peroneal (r=0.85-0.88) and tibial nerve amplitudes (r=0.88), suggesting strong clinical relevance of the observed electrophysiological improvements (Table 2).

**Table 2:** The calculated effect sizes indicate a large magnitude of neurophysiological improvement despite small sample size

Nerve location	Z	p-value	Effect size (r)
M wave proximal – n. peroneus	-3.298	<0.001	0.85
M wave distal– n. peroneus	-3.415	<0.001	0.88
M wave proximal– n. tibialis	-3.408	<0.001	0.88
M wave distal – n. tibialis	-3.409	<0.001	0.88

## Discussion

This study demonstrated statistically significant improvements in peroneal and tibial M-wave amplitudes following three weeks of combined ALA supplementation and proprioceptive rehabilitation in patients with chronic low back pain (CLBP). These findings suggest measurable neurophysiological changes in peripheral motor conduction associated with integrative therapy.

The large effect sizes observed in this study (r ranging from 0.85 to 0.88) indicate a strong magnitude of neurophysiological change despite the small sample size. These findings support the potential clinical significance of the combined intervention and justify further controlled investigation.

Chronic low back pain is increasingly understood as a condition involving both mechanical and neurophysiological components, including altered motor control and peripheral nerve dysfunction [2,16]. Persistent nociceptive input may result in impaired motor unit recruitment and neuromuscular coordination, particularly in patients with chronic radicular involvement [17]. Reduced M-wave amplitude may reflect axonal compromise or decreased motor unit activation. The observed increase in amplitude following intervention suggests improved peripheral motor excitability.

Alpha-lipoic acid has well-documented antioxidant and anti-inflammatory properties [18]. It has demonstrated improvement in nerve conduction velocity and neuropathic symptoms in patients with diabetic neuropathy [19, 20]. Oxidative stress has also been implicated in intervertebral disc degeneration and neural tissue dysfunction [21]. Therefore, metabolic modulation through ALA supplementation may partially explain the electrophysiological improvements observed in this cohort.

Proprioceptive deficits are consistently reported in CLBP populations [2,22]. Altered trunk muscle activation patterns and delayed recruitment of deep stabilizers contribute to spinal instability and pain persistence [23]. Sensorimotor training programs, including instability-based rehabilitation approaches, have demonstrated beneficial effects on motor control and functional outcomes [24]. The Huber system provides controlled multiplanar instability and proprioceptive stimulation, potentially enhancing neuromuscular activation and central motor integration.

The integrative therapeutic model used in this study addresses both biochemical and functional aspects of CLBP. Pharmacological modulation may improve neural metabolic conditions, while structured proprioceptive training enhances motor coordination. The combined intervention was associated with objective electrophysiological improvement, supporting further investigation of this multimodal strategy.

However, several limitations must be acknowledged. The small sample size and lack of a control group limit causal interpretation. The short follow-up period prevents conclusions regarding long-term effects. Additionally, functional clinical outcomes such as pain intensity (visual analog scale; VAS) or disability (Oswestry Disability Index; ODI) were not correlated with electrophysiological findings, which would strengthen clinical applicability.

Future randomized controlled trials with larger samples and longer follow-up periods are necessary to validate these findings and clarify the clinical relevance of combining alpha-lipoic acid supplementation with proprioceptive rehabilitation in chronic low back pain.

## Conclusions

The combination of alpha-lipoic acid supplementation and proprioceptive rehabilitation using the Huber exercise system was associated with significant improvements in motor nerve conduction parameters in patients with chronic low back pain. These findings support the concept of integrative therapy targeting both neurochemical and sensorimotor mechanisms. Further randomized controlled studies are required to determine long-term clinical relevance and establish evidence-based treatment protocols.
